# Primary immunodeficiency-related bronchiectasis in adults: comparison with bronchiectasis of other etiologies in a French reference center

**DOI:** 10.1186/s12931-019-1242-4

**Published:** 2019-12-04

**Authors:** Hélène Goussault, Hélène Salvator, Emilie Catherinot, Marie-Laure Chabi, Colas Tcherakian, Alexandre Chabrol, Morgane Didier, Elisabeth Rivaud, Alain Fischer, Felipe Suarez, Olivier Hermine, Fanny Lanternier, Olivier Lortholary, Nizar Mahlaoui, Philippe Devillier, Louis-Jean Couderc

**Affiliations:** 1 Service de Pneumologie, Hôpital Foch, Suresnes, France; 2 Laboratoire de Pharmacologie Respiratoire UPRES EA 220, Universite Paris Saclay, Versailles, France; 3 Faculté des Sciences de la Santé Simone Veil, Université Versailles-Saint-Quentin-en-Yvelines, Université Paris Saclay, Versailles, France; 4 Service de Radiologie, Hôpital Foch, Suresnes, France; 5 CEREDIH, Centre de Référence des Déficits Immunitaires Héréditaires, Hôpital Universitaire Necker-Enfants Malades, APHP, Paris, France; 6 Service d’Immunologie-Hématologie et Rhumatologie Pédiatrique, Hôpital Universitaire Necker-Enfants Malades, APHP, Paris, France; 7 Institut Imagine, INSERM U1163, Université Paris Descartes–Sorbonne Paris Cité, Paris, France; 8 Collège de France, Paris, France; 9 Service d’Hématologie Adulte, Hôpital Necker-Enfants Malades, Assistance Publique-Hôpitaux de Paris, Paris, France; 10 Service des Maladies Infectieuses et Tropicales, Centre d’Infectiologie Necker Pasteur, Hôpital Necker-Enfants Malades, APHP, Université de Paris, Paris, France

**Keywords:** Bronchiectasis, Primary immunodeficiency, Common variable immune deficiency, Immunoglobulins

## Abstract

**Background:**

Bronchiectasis is a heterogeneous disease depending on etiology. It represents the most frequent non-infectious pulmonary complication of primary immunodeficiencies (PID). We investigated whether bronchiectasis associated with PID had a distinct course in comparison to bronchiectasis of other causes.

**Methods:**

Retrospective single-center study of adult patients diagnosed with non-cystic fibrosis bronchiectasis with more than 5 years of follow-up and at least 4 pulmonary functional tests available at one year apart. They were divided into three groups: PID- related bronchiectasis, idiopathic/post infectious-related bronchiectasis and other causes of bronchiectasis. Respiratory functional data and clinical outcomes were compared.

**Results:**

Of 329 patients with bronchiectasis diagnosed in Foch Hospital (Suresnes, France), 98 patients fulfilled the selected criteria (20 PID-related cases, 39 idiopathic or post-infectious cases, and 39 cases with other causes). Median time of follow-up was 9.5 years. Groups were similar concerning initial characteristics (female 70.4%, never smokers 59.2%, mild severity bronchiectasis according to the FACED score and median FEV1 at diagnosis 73.5% predicted values [Q1–Q3: 53.75–90.5]), except PID patients who were younger (median age of 51.5 vs 62 years, *p* = 0.02). Eighty-five percent of PID patients received immunoglobulin substitution (median trough level was measured at 10.5 g/dl [10;10.92]). Global median FEV1 annual decline was 25.03 ml/year [8.16;43.9] and 19.82 ml/year [16.08;48.02] in the PID patients group. Forty-five percent of patients had bacterial colonization, pneumoniae occurred in 56% of patients and median exacerbation annual rate was 0.8 [0.3–1.4]. Hemoptysis occurred in 31.6% of patients. Global mortality rate was 11.2%. We did not record any significant difference for all clinical and functional outcomes between patients with PID and other etiologies. The median decline in FEV1 was similar in the three groups.

**Conclusions:**

The course of PID-related bronchiectasis was similar to bronchiectasis of other causes. Provided that patients receive immunoglobulin replacement, the course of PID-related bronchiectasis seems to be independent of the underlying immune disorder.

## Background

Bronchiectasis is a chronic respiratory disease, defined radiologically by the presence of permanent bronchial dilatation on high resolution chest computerized tomography.

In recent studies, investigations led to an underlying cause in approximately 40 to 60% of patients [1–6]. Determination of an etiology is important, as it could result in specific care reported in 13% of patients [5].

Primary immunodeficiencies (PID) account for 1–17% of etiologies of patients with bronchiectasis [1–3]. The improvement in their management, mostly immunoglobulin substitution, has allowed us to decrease the prevalence of bacterial pneumoniae and mortality [7–9]. However, bronchiectasis remains the most frequent non-infectious respiratory complication of PID, making its physiopathology unclear and unrelated to the occurrence of previous bacterial pneumonia [10–13].

In bronchiectasis, some factors have been identified as having a poor prognostic value: extension of bronchiectasis [14], chronic obstructive pulmonary disease (COPD) [14] and *Pseudomonas aeruginosa* colonization [15]. However, little is known about mean-term outcome and changes in pulmonary function. The aim of our study was to compare initial characteristics, complications and prognosis of PID-related bronchiectasis with a large cohort of patients with bronchiectasis due to other causes.

## Material and methods

The study received a favorable opinion from the research protocol evaluation committee of the “Société de Pneumologie de Langue Française” (CEPRO 2018–018).

### Patients

#### Baseline findings

We performed a retrospective study of the medical records of all adult patients (> 18 years old) diagnosed with bronchiectasis in the Department of Respiratory Medicine of Foch Hospital, Suresnes, France, between 1984 and 2012. Patients selected for the study were those with more than 5 years of follow-up and with at least four lung function tests available at one year apart.

All data were collected between November and December 2017.

Standard management at the diagnosis of bronchiectasis in our center includes determination of blood cell count; liver and kidney functional tests; detection of rheumatoid factor and anticitrullinated protein antibodies, antinuclear antibodies and anti-neutrophil cytoplasmic antibodies (ANCA); HIV serologic test; serum protein electrophoresis, serum IgG, IgA and IgM levels, serum IgG subclass levels, serum IgE level; detection of *aspergillus* precipitins and aspergillus serologic test; sputum microbiologic examination and culture for bacteria, fungi and mycobacteria. In cases of an unusual clinical presentation or infection with a rare pathogen, we sometimes prescribe additional, targeted immunological analyses. Tests for cystic fibrosis (a sweat chloride assay and/or genetic testing) and for primary ciliary dyskinesia (ciliary ultrastructure analysis or genetic testing) were performed if there was any clinical suspicion (i.e. sterility, diabetes mellitus, sinonasal disease in patients under 40 years of age, situs inversus, or a family history of bronchiectasis). This approach is in line with the guidelines published by the British Thoracic Society [16]**.**

Patients were divided into three groups: primary immunodeficiency; idiopathic and post infectious related bronchiectasis and patients with all other etiologies of bronchiectasis.

Bronchiectasis was defined as of post-infectious origin if there was consistent personal medical history such as pneumonia during childhood or severe whooping cough and no other cause. Bronchiectasis with no cause found at the end of the investigations was defined as idiopathic. Patients with cystic fibrosis (CF) and with traction bronchiectasis related to an interstitial pneumonitis were excluded.

Age at diagnosis (defined by the date of first medical document confirming bronchiectasis), gender, smoking habits (active, former and never smoked), Body Mass Index (BMI), arterial hypertension, gastroesophageal reflux (symptoms or 24-h gastroesophageal pH monitoring), osteoporosis, diabetes mellitus, treatment by statins, initial severity of bronchiectasis, and results of lung function tests were collected. Bronchiectasis severity was evaluated using the FACED score (including the following variables: FEV1% predicted, age, chronic colonization by *Pseudomonas aeruginosa*, radiological extent of the disease, and dyspnea) [17]. The thoracic-CT scans were scored by a trained thoracic radiologist, using the Bhalla score [18]. Thoracic-CT scans were performed in inspiration, with slide sections of 1.5 or 3 mm thickness. Chronic bronchial colonization was considered when at least two sputum cultures were positive for the same pathogen in an otherwise healthy patient with no worsening of his/her respiratory symptoms and no new chest radiological images.

#### Follow-up study

We recorded any prescription of immunomodulatory treatment, including: immunoglobulin substitution, azithromycin (at anti- inflammatory doses, for more than three consecutive months) or omalizumab.

We noted values of respiratory functional test (PFT). At least four PFT results at one year apart were recorded for each patient. Obstruction was defined as FEV1 on vital capacity rate (VC) < 70%. Distension was defined as residual volume (RV) on total lung capacity rate (TLC) > 30%.

We identified each of the following events:

- Respiratory exacerbations, defined as a requirement for antibiotics due to one or more of the following symptoms: increasing sputum volume, worsening sputum purulence, worsening dyspnea, fever, hemoptysis, increased fatigue/malaise.
- Pneumoniae, defined as the presence of radiological opacities associated with signs of exacerbation
- Hemoptysis with or without arterial embolization
- Admission and admission into the intensive care unit (ICU)
- *Pseudomonas aeruginosa* and *Aspergillus sp.* colonization (defined as at least two results of sputum culture separated by at least 3 months in one year)
- Non-tuberculous mycobacterial infection.
- Others: death, cardiovascular disease (myocardial infarction or stroke).

### Statistical analysis

Statistical analyses were performed with GraphPad Prism 7, using the Kruskal Wallis test for non- parametric values and Chi-squared test for contingencies. For all analyses, *p* < 0.05 was considered statistically significant. For demographic and clinical variables, we presented data as median with interquartile range [IQR] for continuous variables and number with percentage for categorical variables.

Lung function change was defined as the difference between first and last tests available. The same method was used for the Bhalla score. In order to take account of differences in follow-up durations and numbers of visits from one patient to another, linear regression (no weighting) was used to find the best-fit value of the slope for FEV1 in each group (expressed as the median [IQR]).

Rate of exacerbation and/or admission was calculated, for each patient, using the number of exacerbations/admissions divided by the duration of follow-up (in year).

In order to identify factors associated with a greater decline in FEV1, univariate and multivariate analyzes were performed from main initial characteristics of the patients, using the online pvalue software (www.pvalue.io).

## Results

Of the 329 adult patients diagnosed with bronchiectasis in our Department between 1984 and 2012, 98 patients fulfilled the study criteria.

### Etiologies of bronchiectasis

**Fig. 1:**
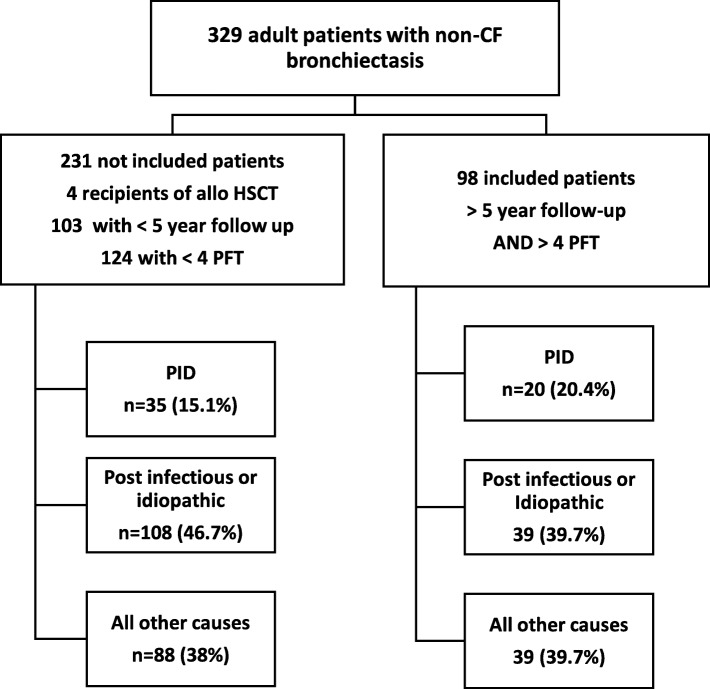
Flow chart. 329 Adult patients (> 18 years old) diagnosed with non-CF bronchiectasis were retrospectively identified. In order to evaluate clinical and functional course, only patients with an available medical follow up of more than 5 years and at least 4 pulmonary functional tests were selected. Finally, patients were divided into three groups according to the cause of bronchiectasis: PID, idiopathic/post-infectious and all other causes. PID: Primary Immunodeficiency; HSCT: Hematopoietic Stem Cell Transplantation; PFT: Pulmonary Functional Test

The proportion of etiologies was similar between the 98 analyzed patients and the 231 other patients with a shorter follow-up time who were not included (See Flow chart, Fig. 1).

Among the 98 included patients, twenty-three (23.5%) had post-infectious bronchiectasis, and 16 (16.3%) idiopathic bronchiectasis.

Twenty (20.41%) had PID: 13 Common Variable Immune Deficiency (CVID), three IgG-2 subclass deficiency, one dyskeratosis congenita, one hyper IgE syndrome (STAT3 dominant negative loss of function deficiency) and one patient with interferon-γ receptor and interleukin-12 deficiency, one patient each.

The last 39 patients (39.8%) were suffering from other etiologies including:

- Systemic diseases in twelve patients (12.3%): four rheumatoid arthritis, one atrophic polychondritis and one Behçet’s disease; six patients had ANCA vasculitis: three with bactericidal/permeability increasing protein antibodies, two with anti-myeloperoxidase antibodies and one with anti-Proteinase 3 antibodies.
- We found non-tuberculous mycobacteria infection in five patients (5.1%): three *Mycobacterium avium complex*, one *Mycobacterium kansasii*, and one *Mycobacterium shimoidei*.
- Chronic respiratory obstructive disease was observed in six patients (asthma: 3; COPD: 3).
- Heterozygous cystic fibrosis transmembrane conductance regulator (CFTR) gene mutation without any other CF manifestation were observed in 4 patients (4.08%)
- 12 other patients each suffered from another distinct disease, including: previous radiotherapy, renal transplantation, Marfan’s disease, HIV infection, primary ciliary dyskinesia, Kartagener’s disease, secondary hypogammaglobulinemia, lymphocytic bronchiolitis, bronchopulmonary dysplasia, alpha-1 anti-trypsin deficiency, allergic broncho-pulmonary aspergillosis, tracheo-bronchomalacia

### Demographic and baseline characteristics (Table 1)

Patients were in majority female (70.4%). The global median age was 59 years [47.75; 68.25]; PID patients were slightly younger than patients with idiopathic and post infectious-related bronchiectasis (51.5 years [34.5; 63.3] vs 62 [56; 70], *p* = 0.02). Most patients (59.2%) had never smoked, with no significant difference between groups. Patients of the idiopathic and post-infectious group were more likely to have high blood pressure than those of the PID group (*p* = 0.04). There was no statistical difference for other comorbidities.

**Table 1:** Initial clinical data

	Primary immunodeficiencies (*n* = 20)	Idiopathic and post infectious (*n* = 39)	Other etiologies (n = 39)	All (*n* = 98)	*P* value
Age (years)	51.5 [34.5–63.25]*	62 [56–70]*	59 [42–68]	59 [47.75–68.25]	0.033
Sex Female/Male	15 / 5	27 /12	27 / 12	69 /29	0.88
BMI (kg/m^2^)	20.3 [18.25–22.53]	22.9 [19.9–26]	22 [19.8–25.9]	21.8 [19.4–25.33]	0.067
Smoking status					0.5
Never	10 (50%)	23 (59%)	25 (64.1%)	58 (59.2%)	
Former	7 (35%)	14 (36%)	9 (23.1%)	30 (30.6%)	
Current	3 (15%)	2 (5%)	5 (12.8%)	10 (10.2%)	
Comorbidities					
Diabetus mellitus	1 (5%)	5 (12.8%)	4 (10.3%)	10 (10.2%)	0.64
Gastroesophageal reflux	7 (35%)	13 (33.3%)	20 (51.3%)	40 (40.8%)	0.23
Arterial hypertension	6 (30%)*	23 (59%)*	12 (30.8%)	41 (41.8%)	0.035
Osteoporosis	2 (10%)	7 (17.9%)	10 (25.6%)	19 (19.4%)	0.34
IgG initial level (g/l)	8 [6.62;10.75]*	11 [10.5;11.5]*	11 [9.5;13.5]*	11 [9.5;12]	0.0001
Trough level (g/l)	10.5 [10;13]				

BMI: Body Mass Index

Results expressed as median with interquartile range

Most PID patients (85%) were receiving immunoglobulin substitution (70.6% intravenous immunoglobulins and 29.4% subcutaneous Ig) (Table 2). This result is in accordance with the guidelines for the management of these patients [19]. The prescribed posology of immunoglobulin was in median 0.68 g/kg/month [0.56;0.92] and median trough level was measured at 10.5 g/dl [10;10.92]. Seven patients (17.9%) among the group “bronchiectasis from other etiologies” were also receiving immunoglobulins because of hypogammaglobulinemia secondary to various immunosuppressive treatments. Azithromycin was given to 52.04% of patients without difference between groups (*p* = 0.78). Most patients had mild severity bronchiectasis according to the FACED score (0–2), with no difference between groups (*p* = 0.25) (Table 3). Median FEV1 at diagnosis was 73.5% of predicted value [53.8; 90.5] and 64.3% of the global population had an obstructive syndrome (FEV1/FVC < 70%), without difference between groups (*p* = 0.53). There were more patients with lung distension in PID group than in the idiopathic and post infectious group (100% vs 76.2%, *p* = 0.02).

**Table 2:** Treatments

	Primary immunodeficiencies (*n* = 20)	Idiopathic and post infectious (*n* = 39)	Other etiologies (n = 39)	All (*n* = 98)	P value
Azithromycin	9 (45%)	21 (53.9%)	21 (53.9%)	51 (52%)	0.78
Omalizumab	0*	4 (10.3%)	7 (17.9%)*	11 (11.2%)	0.044
Immunoglobulin substitution	17 (85%)^£^	1 (2.6%)^£^	7 (17.9%)^£^	25 (25.5%)	< 0.001
Inhaled treatments	10 (50%)	26 (66.7%)	27 (69.2%)	63 (70.4%)	0.32
Corticosteroids	6 (30%)	17 (43.6%)	19 (48.7%)	42 (42.9%)	0.39
Long-acting β-agonist	9 (45%)	26 (66.7%)	25 (64.1%)	60 (61.2%)	0.24
Anticholinergic	4 (20%)	7 (17.9%)	9 (23.1%)	20 (20.4%)	0.85
Triple association	2 (10%)	4 (10.3%)	4 (10.3%)	10 (10.2%)	0.99
Statin use	1 (5%)	5 (12.8%)	10 (25.6%)	16 (16.3%)	0.95

**Table 3:** Initial respiratory data

	Primary immunodeficiencies (n = 20)	Idiopathic and post infectious (n = 39)	Other etiologies (n = 39)	All (n = 98)	P value
Lung function					
FEV1 (L)	1.89 [1.59–2.56]	1.56 [1.16–2.09]	1.68 [1.28–2.34]	1.73 [1.28–2.20]	0.22
FEV1 (% predicted)	75.5 [57.25–92.5]	73 [54–90]	66 [51–92]	73.5 [53.75–90.5]	0.53
FEV1/VC (%)	67 [55–76.25]	62 [52–75]	64 [55–71]	64 [55–74]	0.71
FEV1/VC < 70%	12 (60%)	24 (61.5%)	27 (69.2%)	63 (64.3%)	0.70
RV/TLC > 30%	20 (100%)*	30 (77%)*	35 (89.7%)	85 (86.7%)	0.002
Bhalla score	9 [6;11]	9 [7;11]	9 [7;12]	9 [7;11]	0.55
FACED score					0.6
Mild (0–2)	16 (80%)	23 (59%)	27 (69.2%)	66 (67.3%)	
Moderate (3–4)	3 (15%)	12 (30.8%)	9 (23.1%)	24 (24.4%)	
Severe (5–7)	1 (5%)	4 (10.3%)	3 (7.7%)	8 (8.3%)	

FEV1: Forced Expiratory volume in 1 s; VC: Vital Capacity; RV: Residual Volume; TLC: Total Lung Capacity. Results expressed as median with interquartile range

### Outcomes and follow-up (Table 4)

The overall median follow-up time was 114 months [84.5; 153]. There were no significant intergroup differences in the length of follow-up or the number of visits including a pulmonary functional test (Table 4).

**Table 4:** Outcome

	Primary immunodeficiencies (n = 20)	Idiopathic and post infectious (n = 39)	Other etiologies (n = 39)	All (n = 98)	P value
Follow-up time (months)	110 [87.2;140.5]	122.5 [83;156.2]	114 [79.5;155.5]	114 [84.5;153]	0.92
Number of visits (mean **±** SD)	9.4 ± 3.7	9.4 ± 6.5	10.5 ± 5.4	9.8 ± 5.5	0.44
Admission (days/y)	0.45 [0.05;4.675]	1.4 [0.7;4.2]	1.6 [0.3;6]	1.35 [0.3;5.125]	0.4
Admission in ICU	1 (5%)	9 (23.1%)	7 (17.9%)	17 (17.4%)	0.22
Cardiovascular events	0	3 (7.7%)	3 (7.7%)	6 (6.1%)	0.44
Hemoptysis	4 (20%)	14 (35.9%)	13 (33.3%)	31 (31.6%)	0.44
Exacerbation rate (/y)	0.8 [0.525;1.475]	0.6 [0.25;1.4]	0.8 [0.3;1.5]	0.8 [0.3;1.4]	0.51
Pneumoniae	12 (60%)	19 (52.8%)	24 (61.5%)	55 (56.1%)	0.48
Chronic colonization	5 (25%)*	22 (56.4%)*	17 (43.6%)	44 (44.9%)	0.022
*Aspergillus*	2 (10%)	7 (17.9%)	9 (23.1%)	18 (18.4%)	0.47
*Pseudomonas aeruginosa*	4 (20%)^§^	18 (46.2%)^§^	11 (28.2%)	33 (33.7%)	0.05
NMT infection	1 (5%)	7 (17.9%)	6 (15.4%)	14 (14.3%)	0.39
Lung function					
FEV1 variation (mL/y)	−19.82 [−48.6;−16.1]	−22.86 [−36.7;−4.1]	−28.44 [−49.2;−11.2]	−25.03 [−43.9;−8.2]	0.437
FEV1 variation (%/y)	−1.345 [− 2.5;−0.48]	−1.08 [− 1.85;0.34]	−1.5 [− 2.47;−0.15]	−1.31 [− 2.35;−0.10]	0.48
FEV1/VC variation (%/y)	−0.605 [− 1.44;0.46]	−0.3 [− 1.25;0.39]	−1.00 [− 1.75;−0.27]	−0.55 [− 1.47;0.24]	0.09
RV/TLC variation (%/y)	1.14 [0.15;2.5]	0.85 [−0.51;2.6]	1,19 [−0.44;36]	1.1 [− 0.06;2.7]	0.81
Bhalla score variation(%/y)	0.98 [0;2.5]	1 [0;2.86]	0.96 [0.52;2.77]	0.95 [0;2.74]	0.7
Deceased	2 (10%)	5 (12.8%)	4 (10.3%)	11 (11.2%)	0.95
Respiratory cause	1 (50%)	4 (80%)	3 (75%)	8 (72.7%)	0.72

ICU: Intensive Care Unit; NMT: non-tuberculous Mycobacterial infection; FEV1: Forced Expiratory Volume in 1 s; VC: Vital Capacity; RV: Residual Volume; TLC: Total Lung Capacity. Results expressed as median with interquartile range. When significant differences were reached, they are shown with a superscript note between the tested groups

### Pulmonary function tests

The median [IQR] overall annualized decline in FEV1 was 25.03 mL [8.16; 43.9], corresponding to an annual decrease of 1.31% [2.3; 0.1] in the baseline value. In the PID-related group, the median [IQR] decline in FEV1 was 19.82 ml/year [16.08;48.6]. No statistical difference was observed between the three groups for FEV1 variation during the follow up (*p* = 0.437) (Table 4). In a univariate analysis, factors associated with a significant decline of FEV1 (> 22 ml/year) were a FACED score more than 3 (OR 1.084 [IC95%: 1.013;1.33], *p* < 0.01) and a high mMRC score (OR 1.62 [1.37;1.96], p = 0,044). In a multivariate analysis, only a FACED score of 3 or 4 was still relevant (OR 1.047 [1.004;1.29], p < 0.01).

### Respiratory complications

Chronic bacterial colonization was found in 44% of patients: 33.7% had *Pseudomonas aeruginosa* and 18.4% *Aspergillus sp* (Table 4). Patients from the group “idiopathic and post- infectious bronchiectasis” were more likely to have bacterial bronchial colonization than patients with PID (56.4% vs 25%, *p* = 0.022), especially colonization with *Pseudomonas aeruginosa* (46.2 vs 20%, *p* = 0.049). Rate of exacerbation was not statistically different between groups*,* with a global median of 0.8 exacerbation per year [0.3;1.4] (*p* = 0.51). Most exacerbations (70%) occurred during autumn or winter. Pneumonitis occurred in 60% of patients in PID group, with no statistical difference with other patients (*p* = 0.48). Pathogen identification was not achieved in most cases (59.6%). When recorded, *Pseudomonas aeruginosa* was the most frequently identified (42.1%). There were 11 (29%) infections with *Hemophilus influenzae* and 6 (15.8%) with *Streptococcus pneumoniae*.

Global median duration of admission was 1.35 days per patient per year [0.3;5.1], without significant difference between groups (p = 0.4).

Intensive care admission for any respiratory complication was necessary for 17.4% of patients, but only for 5% in the PID group, without reaching significant difference (*p* = 0.22).

Hemoptysis occurred in 31 patients (31.6%), including four patients in the PID group (20%). Broncho-arterial embolization was required in five cases. Among them, four cases (20%) occurred in PID patients, 14 (31.6%) in post infectious/idiopathic bronchiectasis and 13 (33.3%) in patients with bronchiectasis of other causes. No patient deceased from hemoptysis. None required pulmonary surgery.

### Mortality

Eleven patients (11.2%) died: eight from bronchiectasis exacerbation, three from malignancies (non-pulmonary lymphoma, breast and gastric cancer respectively); mortality rate was similar in the three groups. Of note, the patients suffering from lymphoma belonged to the PID group. No patient died due to a cardiovascular cause.

Deceased patients initially presented a higher initial FACED score (27% patients with a score 5 to 7 vs 3.4%, among survivors *p* < 0.01; 36% with a score 3–4 vs 15% in survivors, p < 0.01) and a lower FEV1 (1159 ± 673 vs 1888 ± 709 ml, p < 0.01). However, comorbidities and smoking status were equivalent. No patient received a lung transplant nor was registered on the waiting list.

## Discussion

We report here, to our knowledge, the first study on PID-related bronchiectasis compared with a large panel of patients with bronchiectasis of other etiologies.

Because all the patients were managed by the same team, it rules out the differences which occur frequently in retrospective multicentric studies. A second point of interest of our study is its long follow up period, with a median time close to 10 years, most previous reported series having a shorter follow up time [14, 20].

Initial data were quite similar between the three groups, except that PID patients were younger. This has already been previously noted with an age in majority less than 50 years in patients with PID-related bronchiectasis [5].

In six recent series of patients with bronchiectasis (1–6), the percentage of PID patients varied from 1 to 17%. This percentage is higher than in previous studies because of an improvement in PID detection [21–23]. We reviewed a relatively high proportion of patients with PID within our cohort because we are a Reference center for the management of adults with PID and related respiratory diseases.

As PID diagnosis results in specific management, it is important that physicians perform an immunological evaluation of all patients with bronchiectasis. During the same study period, 167 patients were seen in our Department for PID-related pulmonary manifestations. Bronchiectasis were observed in 55 patients (33%), mainly in CVID (24 patients). Bronchiectasis is known as the first non-infectious manifestation of CVID, reported in 27 to 79% of patients [10–13]. Patients with CVID should be systematically investigated for bronchiectasis because bronchiectasis is generally isolated whereas granuloma, cytopenia and enteropathy form a set of interrelated features [11].

The prevalence of bronchiectasis is lower in patients with Hyper IgM syndrome than patients with CVID or agammaglobulinemia [10]. Accordingly, patients with Hyper IgM syndrome have a significantly lower risk of non-typeable *Hemophilus influenzae* carriage (RR 0.39; 95% CI, 0.21–0.63) [24]. In CVID patients from the European registry, median IgM level was significantly lower in patients with bronchiectasis than others (0.18 g/L vs 0.26 g/L respectively) [11]. This may suggest a protective effect of IgM on bronchial disorders.

The hyper IgE syndrome with STAT3 dominant negative loss of function deficiency is another PID in which bronchiectasis is also frequently reported (37.5%) [25]. Its course is very peculiar, with rapid development of saccular or cystic bronchiectasis after an infectious event [25]. Physicians should analyze serum IgE level in all patients with bronchiectasis and look for extra-pulmonary manifestations that could be related to hyperIgE syndrome. A clinical score has been established by the National Institute of Health [26] to guide molecular analysis. Chronic granulomatous disease, now an adulthood disease, may be rarely a cause of bronchiectasis [27].

Pneumonitis occurred in 60% patients with PID and exacerbation rate (0.8 per patient per year) were similar to what has been observed in other series (7–9). Although bacterial colonization was less frequent in patients with PID (probably due to the immunoglobulin replacement therapy), the frequencies of pneumonitis and exacerbations were similar in the three groups. This may be related to the viral nature of the exacerbations, with viruses observed in 56% of episodes, as immunoglobulins are only of minor importance in defense against respiratory viruses [28].

Annual decline of FEV1 was calculated at a median of 25.03 ml/y [8.16;43.9] for all causes of bronchiectasis. This rate was lower than in the study of Buscot et al. [29], reporting a median decrease of 30 mL per year in 72 patients with non-CF bronchiectasis. This difference could be explained by the fact that our patients had less severe disease at diagnosis (FEV1 73.5% in our cohort vs 57% of predicted values in the one of Buscot et al. [29]). Particularly in PID-related bronchiectasis, the median decline of FEV1 was calculated at 19.82 mL per year [16.08; 48.6] in our study. This is also lower than the one reported by Chen et al. in 37 Australian patients with CVID or X-linked agammaglobulinemia receiving immunoglobulin therapy; they calculated, over a 7.6-year-long follow-up, a mean annualized decline of FEV1 of 45 mL/y [30]. It is noteworthy that the reported trough level of IgG was lower in this study, at a mean of 7.7 g/l. However, in another study, Hurst et al. enrolled 33 patients with primary antibody deficiency syndromes (all using immunoglobulin replacement) and calculated a median annual rate of FEV1 decline of 28.2 ml/year which was closer to our observation [31]. Otherwise, a short prospective study analyzing functional course in nine CVID patients with bronchiectasis under immunoglobulins replacement, reported no change in pulmonary function test after a 2-year-follow up [32].

The fact that respiratory function keeps declining despite IgG substitution with high trough IgG level above 8 g/dl in patients with predominant antibody deficiency has already been described by our group [10]. It is to be noted that CVID patients with bronchiectasis require higher replacement doses with initial doses of 0,6 g /kg/mo [33] while higher doses of IVIG are associated with a smaller decline in lung function [31]. This has been reported to be due to the possible consequence of an increased catabolism in these patients with bronchiectasis but this has not been clearly demonstrated so far [33]. Doses should be adjusted individually and regularly reconsidered in light of infectious recurrences and of occult bronchiectasis occurrence [34]. This observation is in concordance to what was observed in our PID-related bronchiectasis patients who were prescribed in median 0.68 g/kg/month immunoglobulin and presented relatively high trough levels of Ig (10.5 g/dl in median). High-titer Ig replacement therapy meant that the rate of FEV1 decline in patients with PID-related bronchiectasis was the same as in patients with bronchiectasis due to other causes.

Azithromycin is frequently used for management of patients with bronchiectasis as it reduces exacerbations [35, 36]. Azithromycin has anti-inflammatory properties by decreasing neutrophil recruitment and changing macrophage polarization toward an anti-inflammatory profile [37, 38]. Azithromycin also reduces quorum sensing and biofilm formation of *Pseudomonas aeruginosa* and non-typeable *Hemophilus influenzae* biofilms [39–41]. It has been recently suggested that azithromycin reduces the number of exacerbations in PID-related bronchiectasis patients versus patients treated with a placebo [42].

In our study, the mortality rate was 11.2%. Mortality varied, in other studies, from 10 to 30% [14, 20]. Most deaths were related to respiratory complications, as it was in other studies but a major difference is the absence of cardiovascular cause of death observed in our patients although it has frequently been evoked for patients with bronchiectasis of all causes [43]. It could be due to the high proportion in our cohort of patients with PID, which are slightly younger than others.

The physiopathology of bronchiectasis in PID patients remains unclear. It has been suggested that patients with PID-related bronchiectasis have a greater airway and systemic inflammation than non PID patients [31]. Hurst et al. showed a greater systemic and local magnitude of inflammatory response in patients with PID-related bronchiectasis and a relationship between FEV1 decline rate and systemic inflammation (serum IL-6 level) [31]. However, in our study, the lung function decline in PID-related bronchiectasis patients (mostly receiving Ig replacement) was similar to that of patients with other causes of bronchiectasis. This suggests that the functional decline may not be secondary to the PID-related humoral deficiency and associated immune disorders but rather to local bronchial chronic inflammation intrinsically associated with airways dilatation by itself.

One may also evoke, in PID-related bronchiectasis as in other causes of bronchiectasis, many other predisposing factors supposed to be involved in airways inflammation such as mannose-binding lectin gene polymorphism or high serum concentration of TNF alpha [44, 45]. Recently, the role of pulmonary microbiome, the role of matrix metalloproteinases profile and the activation of natural killer cells leading to lung damage [46–48] have been suggested. Other cells may participate in inflammation, such as neutrophils with elastase cathepsin C or the chemokine receptor CXCR2, macrophages through granulocyte-macrophage colony -stimulating factor (GM-CSF) [49].

## Conclusion

Our study shows that bronchiectasis is frequent in PID patients, highlighting the necessity to perform serum electrophoresis, IgG IgA and IgM serum levels, IgG subclasses and antibodies to *Streptococcus pneumoniae* to detect a humoral PID in all adult patients with bronchiectasis. It is necessary to collaborate in a multidisciplinary network with immunologists for the diagnosis and the management of patients with PID-related bronchiectasis.

For a long follow up time, the course of PID-related bronchiectasis was similar to bronchiectasis of other causes, in terms of acute infection, annual functional decline and mortality rate. Thus, provided that patients receive Ig replacement, the course of PID-related bronchiectasis seems to be independent of the underlying immune disorder. These observations highlight (i) the protective effect of Ig replacement therapy on respiratory outcomes in patients with PID-related bronchiectasis and (ii) the importance of regularly monitoring the trough Ig level in these patients. However, Ig replacement therapy did not protect fully against lung involvement, and lung function continued to decline in PID patients to the same extent as in patients with bronchiectasis from other causes – suggesting that this functional decline is due to the chronic inflammation caused by airway dilatation, rather than humoral deficiency.

## Abbreviations

ANCA: anti-neutrophil cytoplasmic antibody
BMI: body mass index
CEREDIH: centre de référence des déficits immunitaires héréditaires
CF: cystic fibrosis
CFTR: cystic fibrosis transmembrane conductance regulator
COPD: chronic obstructive pulmonary disease
CVID: common variable immune deficiency
FEV1: forced expiratory volume in 1 s
ICU: intensive care unit
Ig: immunoglobulin
IVIg: intravenous immunoglobulin
MRC: medical research council
NTM: non-tuberculous mycobacteria
PID: primary immunodeficiency
RV: Residual Volume
TLC: total lung capacity
VC: Vital Capacity
